# Progress in Hematopoietic Stem Cell Transplantation for CIDP

**DOI:** 10.7150/ijms.38363

**Published:** 2020-01-14

**Authors:** Zhen Qin, Qianyi Huang, Jiangying Zou, Lisha Tang, Zhiping Hu, Xiangqi Tang

**Affiliations:** 1 Department of Neurology, The Second Xiangya Hospital, Central South University, Renmin Road 139#, Changsha, 410011, Hunan, China; 2 Healing With Stem Cell Therapy Inc, PO Box 2289, Shawnee Mission, 66201, KS, USA

**Keywords:** Chronic inflammatory demyelinating polyneuropathy, Hematopoietic stem cell transplantation, autoimmune diseases, demyelinating neuropathy

## Abstract

Chronic inflammatory demyelinating polyneuropathy (CIDP) is a kind of autoimmune-mediated inflammation and demyelinating disease. The etiology is mainly related to autoimmune dysfunction. The conventional treatments of CIDP have relied on immunomodulation and inhibition therapies such as adrenal cortex hormone, intravenous immunoglobulin (IVIg) and plasma exchange. Hematopoietic stem cell transplantation (HSCT) is known as a novel therapy for autoimmune disorders, which provides the chance to cure CIDP. More than 70 patients with refractory CIDP have received HSCT. The clinical symptoms and electrophysiological examination results of most patients have been improved. However, the treatment still has risks. This review describes the pathogenesis of CIDP and the current conventional treatments, and highlights the application of HSCT in CIDP, including its efficacy and safety.

## 1. Introduction

### 1.1 Clinical manifestations and diagnosis

CIDP is mainly a group of clinical syndromes. The course of the disease is chronic progression or recurrence lasting more than 8 weeks. It often manifested as symmetrical weakness of the proximal or distal limbs, loss of sensation and tendon reflexes. The main diagnostic methods include neurophysiological test, cerebrospinal fluid and pathological examination. Most patients often have cerebrospinal fluid protein-cell separation. Neuroelectrophysiological test suggests demyelinating lesions. Nerve biopsy shows segmental demyelination and regeneration, even the formation of “onion bulbs-like” changes. A retrospective study by Karine et al. 1 showed that approximately 25% of patients with CIDP in the clinic did not meet the current diagnostic criteria for EFNS/PNS. Electrophysiological, clinical and other evidence is needed if diagnosis is to be made. There are still a small number of patients with atypical clinical symptoms, which can be manifested as autonomic dysfunction. Long et al. 2 reported a case of CIDP manifested as recurrent intestinal obstruction.

### 1.2 Pathogenesis of CIDP

The pathogenesis is still not very clear. Previous clinical case reports show that the onset of CIDP may be related to the induction of specific infections such as hepatitis B virus, EB virus and hepatitis C virus etc. 3, 4, 5, 6. The pathogen may carry elements that are similar in amino acid sequence to self-antigen and act as “molecular mimicry”. The infections may cause immune reactions leading to an autoimmune process. Antigen-presenting cells (APCs) process and present antigens throughout the major histocompatibility complex (MHC) to T cells. Thereafter, cytolytic T cells can directly lyse a target. T helper cells release mediators such as cytokines to activate macrophages, monocytes, and B cells. These immune reactions are also cross-reactive to self and lead to tissue damage. APCs take up antigens released from damaged tissue and initiate a self-specific immune response. Bystander activation is also one of the pathogenesis of CIDP, which is characterized by nonspecific and inaccurate immune response to pathogens, leading to tissue damage 7.

The main targets of antibodies in CIDP are antigens on myelin and Schwann cells 8. At present, the antigens studied more are contactin-1, contactin-associated protein-1 (CASPR1) and neurofascin-155 (NF155) located in the paranode region. Koike et al. 9 have found that the frequency of myelin loops detaching from the axolemma at the terminal of paranode in CIDP patients with positive anti-contactin 1 and anti-NF155 antibodies 10, comparing with negative ones, is significantly increased.

### 1.3 Pathological changes

Infiltration of T lymphocytes 11 and macrophages 12 can be seen by sural nerve biopsy. T cells are activated by antigen and secrete pro-inflammatory cytokines, such as IL-2 13, IFN γ, IL-17 14 and chemokine IP-10, MIP3B 15. Activated macrophages induce up-regulation of endothelial leukocyte adhesion molecule (ECAM-1), intercellular adhesion molecule (ICAM-1) 16, 17 expression on neurovascular endothelial cells. T cells adhere to vascular epithelium through interaction with adhesion molecules, then move along vascular endothelium, and continue to secrete inflammatory regulatory factors (matrix metalloprotein, chemokines and pro-inflammatory cytokines) through the blood-nerve barrier. The destruction of the blood-nerve barrier, making soluble substances such as antibodies more accessible to the endometrium, aggravates the inflammatory response of nerve tissue 18.

Macrophages are also the main inflammatory cells and form colonies around blood vessels in nerves 19. Meanwhile macrophages play a dual role in the whole inflammatory reaction. The detrimental aspect is that macrophages can act as antigen presenting cells to promote autoimmune reaction and secrete pro-inflammatory factors (such as IL-1, IL-12, TNF-a) to regulate the inflammatory reaction process. The favorable aspect is that in the late stage of inflammation, macrophages can promote T cell apoptosis and express anti-inflammatory factors (such as TGF-B1 and IL-10) to terminate the inflammatory process and participate in the proliferation of Schwann cells and regeneration of myelin axons during the disease recovery stage 20.

### 1.4 Conventional treatments

Current treatments that have been validated by clinical randomized controlled trials include corticosteroid, plasma exchange and IVIg. Regarding the study of corticosteroid therapy, in a prospective cohort study in 2012 21 patients were treated with pulse dexamethasone or prednisone daily for a total of 1 to 2 courses of treatment. Thirty-nine patients achieved symptom relief through half of the initial treatment. The median time between the onset and recurrence of symptoms was 17.5 months for dexamethasone and 11 months for prednisone. In a retrospective study in 2018 22, pulse oral dexamethasone (37 patients), pulse intravenous methylprednisolone (21 patients) and daily prednisone (67 patients) were given to confirmed CIDP patients respectively. The overall effective rate of 125 patients was 60%. There was no significant differences among the three treatment methods (P=0.56).

The clinical effect of plasma exchange was confirmed in early clinical trials in 1986 23. In a prospective double-blind trial, 15 patients were treated with plasma exchange and 14 patients were treated with sham treatment. The results showed that the improvement of symptoms and electrophysiology in patients treated with plasma exchange was more significant than that in the sham treatment group (P=0.025). A double-blind, sham-treated control and cross-over clinical trial in 1996 24 showed that compared with the sham-treated group, the plasma exchange group had more obvious improvement in neurological disability score (NDS) (P<0.001) ,clinical grade (CG) (P<0.001) and grip strength (GS) (P<0.003) of neurological functional disability score.

In 2001, Mendell et al. 25 randomly assigned 53 people to IVIg group (30 people) and placebo control group (23 people). After 6 weeks of treatment (two dropouts in placebo group and one in the IVIg group), the average muscle score (AMS) of IVIG group was measured. Compared with the control group, the improvement of IVIG group was significant (P=0.006). Electrophysiological examination results including the latent period of distal ulnar nerve action potential (P=0.005), distal tibial compound muscle action potential (P=0.003), and peroneal nerve conduction velocity (P=0.03) were also significantly improved. Intravenous or subcutaneous injection is safe and effective for both long-term and short-term use 26, 27, 28.

Apart from the above-mentioned first-line treatments, other immunosuppressive and regulatory drugs have also been reported for clinical use and research, such as azathioprine. A clinical randomized trial in 1985 29, 27 patients were divided into prednisone treatment group and prednisone combined azathioprine treatment group. The results showed that there was no significant difference between the two groups. Methotrexate was reported for clinical use in 2005 30, 31, but a randomized controlled trial in 2009 showed that taking 15mg of methotrexate per week had no significant effect. Cyclophosphamide 32, 33, 34, cyclosporine 35, 36, 37 and mycophenolate 38, 39, 40 have been proved to have clinical efficacy by many literature reports, especially for patients with first-line therapeutic resistance. Alemtuzumab was reported to be used clinically in 2005. A case of IVIg-dependent refractory CIDP patient achieved good clinical efficacy. The studies in 2010 showed that the average dosage of IVIg decreased by 26% per month after the use of Alemtuzumab, and the use interval was extended from 22 days to 136 days 41, 42.

The clinical use of interferon (IFN) has been reported 43, 44, 45, but there are cases of CIDP caused by the use of IFN to treat other diseases 46, 47, 48, mainly the complications after IFN is used to treat viral infectious diseases such as HIV and HCV. In 1999, a randomized controlled trial confirmed that IFN-β-1α had no obvious effect on CIDP patients with resistance to first-line treatments 49.

Although there are so many immunosuppressive therapies, they cannot induce long-term, drug-free and symptom-free remission, some patients with CIDP still relapse, even leading to disability and death. Therefore, patients may choose hematopoietic stem cell transplantation as a better treatment option.

## 2. Hematopoietic stem cell transplantation for CIDP

### 2.1 Purpose and Basic Steps of Hematopoietic Stem Cell Transplantation

Hematopoietic stem cell transplantation has been widely used to treat refractory autoimmune diseases, aiming at eradicating autoreactive immune cells and rebuilding a new immune system that is self-tolerant. Relief of clinical symptoms of autoimmune diseases after hematopoietic stem cell transplantation is the result of immune system reconstruction 50. Currently hematopoietic stem cell transplantation is used for refractory CIDP patients. The stem cells can come from their own bone marrow or peripheral blood. According to EBMT (European Society for Blood and Marrow Transplantation) recommendation, cytokine-mobilized peripheral blood stem cells are the best choice for autologous hematopoietic stem cell transplantation because more CD34+ stem cells can be obtained, which is better than bone marrow-derived stem cells for transplantation and can rebuild hematopoietic system faster 51. The general steps of stem cell transplantation include patient selection, flare prevention, mobilization, stem cell collection, conditioning, stem cell infusion, supportive care during neutrophil and lymphocyte reconstruction, and post-transplantation care 52.

### 2.2 Changes of symptoms and neurological score before and after HSCT treatment

From 2002 to 2018, there are about 70 cases of refractory CIDP treated by HSCT worldwide. In general, the relapse frequency of the patients has decreased with different degrees. The electrophysiological examination results have been significantly improved. The symptoms have diminished, the progress of the disease has been effectively controlled, and some patients have stopped immune regulation or inhibition therapy after HSCT.

The earliest reported treatment of CIDP with hematopoietic stem cell transplantation was in 2002. Vermeulen et al. 53 reported a 38-year-old male patient with CIDP for 10 years. The patient suffered from repeated illness without spontaneous remission. Due to serious side effects of immunosuppressive therapy, autologous hematopoietic stem cell transplantation was considered. Before hematopoietic stem cell transplantation, the patient's MRC score was grade 4. There was atrophy of his intrinsic hand muscles and areflexia. He had numbness of his arms and legs and weakness of limbs. He was independent in daily activities. He was treated with autologous hematopoietic stem cell transplantation 10 years after onset, G-CSF+ cyclophosphamide mobilized hematopoietic cells, and the conditioning regimen was BEAM. After HSCT, the patient's symptoms improved, and there was no recurrence within 2 years. The symptoms were only slight numbness of fingertip, but the disease was reported to recur 5 years later.

In 2007, Oyama 54 reported the first patient treated in phaseⅠtrial of autologous HSCT for refractory CIDP, which utilized a nonmyeloablative regimen to avoid potentially lethal regimen-related toxicities. The patient was a 32-years old female, presented with gradual onset of paresthesia, progressive weakness of extremities and paresthesia and twitching of the face and limbs. She was maintained on twice-weekly plasma exchange which led to three episodes of pheresis catheter-related bacteremia and disease exacerbations. Thus, recurrent exacerbations and immune-related complications resulted in referral for HSCT. After HSCT, the symptoms of the patient have diminished, and Rankin function score improved from 4 to 1.

In 2016, R. Kotas 55 reported a patient with CIDP associated with monoclonal gammopathy of undetermined significance (MGUS). The disease began in 2004 with sensory symptoms switched into a classic form with flaccid paraparesis. The patient was treated with immunomodulation and inhibition therapy, but with very frequent relapses. Finally, AHSCT was performed, the frequency of relapses substantially decreased.

From 2002 till now, another 66 patients with refractory CIDP received HSCT treatment. These patients needed to fulfil the definite, probable, or possible EFNS/PNS criteria for CIDP. They were incomplete response or relapse after first-line treatment, and non-response to at least one second-line immunosuppressive drug (methotrexate, azathioprine, cyclosporine, mycophenolate mofetil, etc.). After HSCT, most of them have achieved good clinical efficacy. See Table 1 for disease changes before and after treatment (include neurological function score, symptoms changes, and remission period).

### 2.3 Changes of electrophysiological reexamination after HSCT

Electrophysiological examination is an important method to evaluate the efficacy of HSCT in patients with CIDP. Specific observation indexes include compound muscle action potential (CMAP), sensory nerve action potential (SNAP) and nerve conduction velocity of limbs. The increase in amplitude of action potential and conduction velocity often indicate remission of the disease.

Vermeulen et al. 48 in 2002 reported a case of CIDP reexamined by electrophysiological examination one year after HSCT treatment. Right median nerve: the distal CMAP recovered from 2.1mv to 7.3mv, the latency of nerve terminal action potential recovered from 14.6ms to 12.3ms, and the nerve conduction velocity recovered from 12m/s to 24m/s.

Remenyi et al. 51 reported in 2007 that the electrophysiological examination of CIDP patients before HSCT: Right median nerve MCV 31m/s, MCA 2.2mV, distal latency 11ms, SCV 28m/s, SCA 6.6mV. Eight months after HSCT, the reexamination results showed that MCV 52m/s, MCA 5.3mV and distal latency 3.9 ms. The results were significantly improved compared with those before. The other ulnar nerve, radial nerve, peroneal nerve and tibial nerve were improved regardless of their left and right sides.

In 2009, Mahdi-Rogers et al. 52 reported the HSCT of 6 patients with chronic acquired demyelinating neuropathy, of which 3 were diagnosed as CIDP. One 29-year-old female patients had no obvious changes in electrophysiology after 6 months of HSCT reexamination, but the clinical symptoms continued to improve for 18 months. Besides, electrophysiological changes after HSCT treatment in a 58-year-old patient were better. Latency of right median nerve terminal action potential recovered from 7.6ms to 7.0ms, and motor nerve conduction velocity improved from 38m/s to 41 m/s. The compound action potential of distal muscle recovered from 2.3mV to 6.4mV. Electrophysiological changes were not mentioned in third patient.

In 2013, Press et al. 55 retrospectively studied 11 CIDP patients treated with HSCT. Electrophysiological examination 4 months after transplantation suggested that the median CMAP was 1.84mV with range of 0.1-3.67mV, which was significantly improved compared with the median CMAP of 0.88mV and range of 0.3-2.63mv before HSCT treatment (p < 0.05).

### 2.4 Safety of HSCT

Safety-related indicators of HSCT therapy include adverse reactions, toxic effects and transplant-related mortality (TRM). Adverse reactions and toxic effects often include early adverse reactions and toxic effects (within 100 days after transplantation) and late adverse reactions and toxic effects (after 100 days of transplantation). The former may be related to allergic reaction and side effect induced by immunosuppressive drugs and opportunistic infection after immunosuppression. Common symptoms include fever, infection, abnormal liver function, etc. Bacterial or fungal infection occurs early after HSCT in neutropenia, while long-term hypolymphemia can reactivate latent viruses and other opportunistic infections, thus endangering patients until their immune systems are rebuilt 65. Therefore, all patients should receive broad-spectrum antibacterial, antifungal and anti-herpes prevention at least 100 days after transplantation during aplasia after HSCT. In addition, all patients in neutropenia should prevent the epidemic opportunistic but rapidly fatal bacterium pneumocystis 66. The late adverse reactions usually relate to secondary thyroid diseases, hemocytopenia etc. In HSCT treatment of other autoimmune diseases, late adverse reactions and toxic effects include coagulation abnormalities, myasthenia gravis, etc. These complications may be related to the patient's underlying susceptibility of an “autoimmune-prone” nature, coupled with the immune dysregulation resulting from auto-HSCT and lympho-depleting agents 67.

Compared with autologous hematopoietic stem cell transplantation, allogeneic hematopoietic stem cell transplantation may bring more complications and higher mortality 68. For example, allogeneic stem cell transplantation may cause graft-versus-host disease, which almost affects all tissues and organs. Allogeneic stem cell transplantation also increases the chance of solid tumors 69.

Transplant-related mortality is the most important problem in HSCT. In 2010 Gualandi et al. 70 reported one patient with CIDP died 30 days after ASCT because of pneumonia. In 2017 Kalliopi et al. 71 reported two patients who received autologous peripheral blood stem cell transplantation after the treatment of bortezomib had fatal infections and finally died. However, the specific process of hematopoietic stem cell transplantation is not given in the literature. Pretreatment (including myeloablative and non-myeloablative) is required before HSCT. These treatments require a large number of immunosuppressive drugs, which may lead to complications such as infection or coagulation dysfunction. Reported adverse reactions and toxic effects after HSCT treatment are shown in Table 2.

## 3. Conclusion

CIDP is an autoimmune disease, which is currently considered to be related to cellular and humoral immune abnormalities. Patients are often characterized by loss of sensation and weakness of limbs. Severe cases may even lead to disability or even death. Conventional treatments include immunosuppression and immune regulation, but some patients still have no response to such treatments, so HSCT is used. So far, more than 70 people have been treated with HSCT. HSCT is the only therapy that can induce long-term, asymptomatic remission in refractory autoimmune diseases and a possible new way to solve the problem of autoimmune regulation abnormalities, so that it provides a new approach and method for refractory CIDP. Judging from the effect of current preclinical tests on CIDP, although large-scale clinical RCT tests are lacking, and cases of relapse and complications related to HSCT have been reported, HSCT is safe and feasible in general. The symptoms and neurological score of most patients improved and a longer-term remission period was obtained. The complications after HSCT include toxic and side effects of conditioning drugs, virus and bacterial infection caused by hypo immunity after transplantation, but most of them can be improved by corresponding treatments. In conclusion, HSCT is a viable treatment option for refractory CIDP.

## Figures and Tables

**Figure 1:**
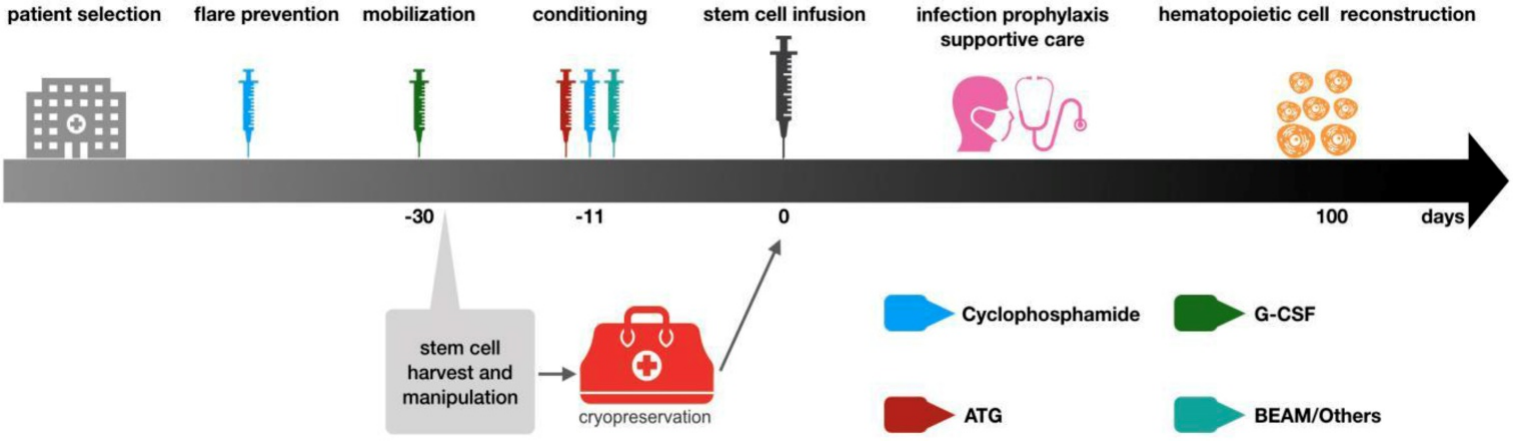
** General procedures of autologous hematopoietic stem cell transplantation.** The above steps are based on EBMT guidelines. The patient selection is mainly aimed at those patients who have received standardized treatment and the condition is still deteriorating. These patients often suffer from functional impair of other organs. Thus, patients should be carefully evaluated before deciding to undergo stem cell transplantation treatment to ensure that HSCT can bring the greatest benefits to patients. After the patient selection, the mobilization will be initiating. The drug used in mobilization is G-CSF, but it may lead to the flare of autoimmune diseases, so cyclophosphamide immunosuppressive therapy will be used before mobilization. The collection of stem cells is generally 4-5 days after mobilization with G-CSF and then the cells are cryopreserved. Before stem cell reinfusion, 1-2 week carries out immune ablation conditioning on patients. Conventional regimens include BEAM and ATG+ cyclophosphamide. After stem cell reinfusion, care should be strengthened to prevent infection. Patients could be discharged when the number of neutrophils recovers and should reexamine regularly.

**Table 1:** Disease Changes Before and After HSCT Treatment

Study (year)	Number	Age (year)	Duration (month)	Symptoms Before HSCT Treatment	Pretreatment protocol /stem cell source	Follow-uptime (month)	Follow-up results
Remenyi et al. (2007) 56	1	26	8	Numbness and fatigue extend to limbs and trunk, vital capacity was decreased to 1100ml, decreases in all sensation modalities, aplastic anemia	Cyclophosphamide+ATG/Allogeneic peripheral blood stem cells	78	Sensation and muscle strength recovered. There was no recurrence within 6.5 years.
Mahdi-Rogers et al. (2009) 57	3	29	21	Flaccid areflexic tetraparesis with marked distal wasting and severe distal sensory loss. MRC sum score: 39	cyclophosphamide+ATG/peripheral blood stem cells.	18	MRC sum score 48, 10m walk time had reduced from 10 to 7 s. Symptoms recurred after 18 months of vaccination.
		72	13	Flaccid areflexic tetraparesis with distal weakness, loss of all sensory modalities to his elbows and knees	cyclophosphamide+ATG/peripheral blood stem cells.	21	MRC sum score fell from 46 to 32. Dependent on a wheelchair. No treatment is required.
		58	84	Symmetrical proximal weakness in upper and distal weakness in lower extremities, mild sensory disturbances, double vision, dysphagia	cyclophosphamide,+ATG/Autologous peripheral blood stem cells	6	MRC sum score improved from 66 to 70, complete remission without any immunomodulating therapy.
Axelson et al. (2008) 58	1	56	12	Bedridden and unable to lift his limbs from the bed	First time: high dose cyclophosphamide/Autologous peripheral blood stem cells Second time: Same as the first time	48	Gait, muscle strength and sensation were normal, tendon reflexes could be elicited. 2 years after the stem cell transplantation the patient relapsed, 3 weeks after second transplantation, muscle strength was normal. Remains in clinical remission after 3 years.
Bregante et al. (2013) 59	1	29	8	Combine with Sjogren's Syndrome. bilateral pleural effusions. Sensory dysfunction weakness of limbs, cannot walk independently	Thiotepa + cyclophosphamide+ G-CSF/Autologous peripheral blood stem cells. Second time: fludarabine+cyclophosphamide/ Allogeneic stem cell	96	6-month-long remission after first HSCT. Relapsed and developed severe aplastic anemia 6 months later. At the 6th year after the second allogeneic HSCT, mucous membrane dryness disappeared, be able to walk independently.
Press et al. (2013) 60	11	Median age: 55	30	Median INCAT score: 6 range: 2-10 Median Rankin score: 4 range: 2-5	Cyclophosphamide (n=7) melphalan (n=1) BEAM (n=3) /Autologous peripheral blood stem cells	Median follow up: 28	28 months after transplantation: Median INCAT score 1 range: 0-5 Median Rankin score 1 range: 0-3
Scheibe et al. (2016) 61	1	39	31	Tetraplegia and respiratory insufficiency	Cyclophosphamide/autologous stem cell transplantation	42	Clinical symptoms were completely relieved within 5 years after transplantation without any treatment.
Barreira et al. (2010) 62	1	46	156	Flaccid paraplegia and wheelchair-bond	cyclophosphamide,+ATG/Autologous peripheral blood stem cells	11	Could stand up and walk130m with aid of walker. Limb muscles strength was graded 4 instead of 3 or less.
Ajroud-Driss et al. (2011) 63	15	Median age:55	Not mentioned	There is no mention in the article.	cyclophosphamide,+ATG/Autologous peripheral blood stem cells	Median follow up: 6	Nine patients are in remission, they are off any treatment, have significant improvement in strength. Four patients are tapering medications. One patient loss follow up and one patient worsened.
Allen JA et al. (2013) 64	32	Mean age:44	Not mentioned	There is no mention in the article.	cyclophosphamide+ATG+methylprednisolone+rituximab/Autologous peripheral blood stem cells	6 (n=27) 12 (18) 24 (12) 36 (8) 48 (4) 60 (3)	Two patients died of pre-existing conditions. Drug free remission was observed in 70% (6month) , 67% (1 year) , 67% (2 year) , 63% (3 year) , 50% (4 year) and 67% (5 year) . Modified rankin scale and SF-36 questionnaire of most patients improved.

**Table 2:** Adverse Reactions and Toxic Effects of HSCT on CIDP

Study (year)	Within 100 days (cases)	After 100 days (cases)
Remenyi et al. 51 (2007)	0	temporary alopecia (1)
Mahdi-Rogers et al. 52 (2009)	severe pneumonia (1) neutropenic fever (1)	0
Axelson et al. 53 (2009)	fever, bronchitis and elevated liver enzymes (1)	0
Bregante et al. 54 (2013)	GVHD After the second allogeneic hematopoietic stem cell transplantation, it was limited to skin only (1)	aplastic anemia Six months after the first autologous stem cell transplantation (1)
Press et al. 55 (2013)	cytomegalovirus (CMV) and reactivation (3) transient CMV colitis (1) Epstein-Barr virus (EBV) reactivation (1) Escherichia coli bacteremia (1) BK-virus-positive hemorrhagic cystitis (2) coagulase-negative Staphylococci bacteremia (1) Klebsiella, Pseudomonas and α-Streptococci septicemias (1) pancreatitis (1)	anemia (1) neutropenia (1) hypothyroid (1) relapsing Clostridium difficile-positive diarrhea (1)
Scheibe et al. 56 (2016)	catheter associated sepsis (1)	0
